# Observations on Exhaustion from the Effects of Heat

**Published:** 1854-07

**Authors:** H. S. Swift

**Affiliations:** Resident Physician of the New York Hospital


Observations on Exhaustion from the Effects of Heat. (*Coup de Soleil.*) By H. S. Swift, M. D., Resident Physician of the New York Hospital.—The following paper will be read with interest. *Coup de Soleil*, as it is generally termed—Sun-stroke—more correctly, *exhaustion from the effects of heat*—is an accident which is becoming frequent in our large cities, and is occasionally heard of in the country. We have seen several cases in Louisville within the last few weeks, during which the thermometer rose to 94, 95, and 97 degrees. A number of fatal cases occurred in the city. Dr. Swift presents, in our opinion, the correct view of the nature and treatment of this affection.

"Owing to the oppressive and long-continued hot weather of the past summer, an unusually large number of cases were admitted to the New York Hospital of what is called *coup de soleil*, or, as now regarded by the profession, extreme prostration produced by exposure to excessive heats, combined, perhaps, with the effect of receiving large draughts of cold water into the system, when overheated.

So prevalent, indeed, was this disease, that at one time it was regarded almost as an epidemic, not only in this, but in neighboring cities. Several cases occurred in the country, where, heretofore, it has seldom appeared. It will be recollected that a large per cent. of the cases were fatal. The report of the City Inspector of this city alone shows 260 deaths from *coup de soleil*, without including many cases designated as "congestion of the brain" and the "effects of cold water."

It is now only five or six years since the nature of this disease was pointed out, and yet the profession, generally, have but vague and indefinite ideas respecting it, and it is a matter of surprise that medical literature is so deficient on this subject. A few short monographs, and a few reported cures, are all that can be found in regard to it. Cases are not so infrequent, nor is this affection so devoid of interest, as this silence would seem to indicate.

I have no new theories to propose, or any new light to throw upon the pathology or the treatment of this disease; the object of this paper is simply to call the attention of the profession to this subject, more especially as the season is now approaching in which we may reasonably expect a return of this "calamity.,'

The term *coup de soleil* as applied to this disease, is a misnomer. It is a popular rather than a professional appellation. All our authors agree that "cerebral apoplexy" is occasionally produced by exposure to the direct rays of the sun. This I regard as true *coup de soleil.* The subject now under consideration is an entirely distinct affection. It is now almost universally admitted to be mere nervous exhaustion produced by protracted and violent exercise in an over-heated atmosphere.

Of the large number of cases observed by me, none were strictly apoplectic, and no lesions were noticed in those which proved fatal, sufficient to account for death. Those two opposite conditions—the "cerebral congestion" and "nervous debility"—require opposite modes of treatment, and should be carefully distinguished from each other.

The subjects of this affection are usually laborers who have been exposed several hours during the day to the direct rays of the sun, the thermometer being over 90°. A great majority of the following cases were foreigners, many of whom had but recently arrived in this country, and who, after the deprivations of a long passage, were ill-adapted to endure great fatigue in so high an elevation of temperature.

The same condition may result after exposure to artificial as well as solar heat. Eleven patients were attacked one morning in the laundry of one of our principal hotels; several were brought to us from a sugar refinery, where, after working several hours in a close and over-heated apartment, they fell down suddenly in a state of insensibility; and we had an opportunity of comparing their symptoms and lesions with those who became exhausted after laboring in the sun, but were unable to satisfy ourselves of any distinction.

Whatever tends to enfeeble the vital powers must be regarded as the predisposing cause. This may result from muscular debility or pre-existing disease. Heat acts as the exciting cause. One patient had suffered for several weeks from an obstinate diarrhœa. He had eaten nothing on the morning of the attack, and, after imprudently walking only a short distance in the sun, fell down insensible. Another patient was suffering at the time of the attack, as we afterwards learned, from the usual *malaise* of fever, and after convalescing from this disease, passed through the ordinary attack of petechial typhus. Still another was in a cachectic condition from the influence of malaria. He was also picked up in the street, and brought to the hospital in an insensible condition. These cases were not included in our report, though they were evidently suffering from this disease at the time of their admission to the hospital.

An attempt has been made to distinguish those cases which are the result of exhaustion merely, and those who have been suddenly seized after drinking large draughts of cold water when overheated either from exposure to the sun or by violent exercise. If such a distinction exists, by far the greater number of cases which fell under my observation would be included in the latter class, though only in a single instance were we able to trace any *immediate* connection. A seaman had been employed, during the day, in the rigging of a vessel, exposed to the direct rays of the sun. At 3 o'clock P. M., he complained of a severe pain in the head and a "sense of sinking within him." After drinking very freely from a bucket of hydrant water, he plunged his head into it, and immediately fell down insensible. Most of the patients had been drinking water freely during the day,—some moderately,—while others had scrupulously avoided it. But a large majority of them were attacked immediately after dinner, when probably large draughts of water were employed.

For this reason I am inclined to believe that the effect of the cold water in these cases is merely to hasten the development of the disease, and that a majority of the cases reported as deaths from "drinking cold water," are really occasioned by "solar exhaustion." Nearly all the patients were exhausted by severe labor, and at their dinner they were in just the condition to suffer from the shock of receiving a large quantity of water suddenly into the system.

Deaths from the effects of cold water are not so frequently met with as is generally supposed. Dr. Dickson, of Charleston, S. C., says: "I have never seen a death from drinking cold water, nor have I been able to obtain any authentic account of such an event having occurred since I have been engaged in the practice of medicine in this city. Yet here, if anywhere, such accidents should occur. Immense quantities of ice and iced fluids are daily consumed here by persons subjected to the several conditions which are regarded as calculated to favor the morbid influence of the agent in the highest degree. The cases described by Rush I believe to have been generally cases of insolation, and that, being sensible of rapidly approaching disease, and at the same time feeling an internal heat, the patients were just procuring relief when overtaken by sudden death." Such, undoubtedly, was the case of the sailor above referred to.

The disease is usually stated to be confined to patients of irregular habits; but only a small proportion—at least less than one-half of the following cases—could be regarded as intemperate, and many of these had restricted themselves during the day to a single glass of ale or brandy.

The premonitory symptoms are usually slight, and of short duration. A laborer may, perhaps, have been employed until a late hour the previous night, and the next morning complains of a slight headache and a general feeling of languor. He takes his breakfast with less relish than usual, but resumes his ordinary duties. But, in the great majority of cases, even these slight symptoms are wanting. They are suddenly seized, while in the performance of their labors, with pain in the head, and a sense of fullness and oppression in the epigastrium, occasionally nausea and vomiting, general feeling of weakness, especially of the lower extremities, vertigo, dimness of vision, and insensibility. Surrounding objects appear of uniform color. In a great majority of cases, this was, so far as could be ascertained, blue or purple. In one instance, everything appeared red; in another, green; and in another, white. One stated that objects retained their natural color, but expressed them as being very beautiful, while to another everything appeared greatly magnified.

This may be regarded as the first stage of the disease. It is usually of short duration. In the milder forms of the disease, the stupor is only momentary. The patient is at first, perhaps, aroused with difficulty, but he gradually regains his consciousness. If, however, the attack is severe, the patient shortly passes into a state of coma. The skin is hot and pungent to the touch, and by actual experiment, according to the observations of Dr. Dowler, the temperature is elevated to 112° Fahr. The pupils are dilated and insensible to light; the breathing hurried and labored; the pulse is sometimes slow and full—sometimes frequent and feeble, though the action of the heart may continue inordinately strong up to the last moment of life.

In the third stage, the symptoms are those of collapse. The pulse becomes more frequent and feeble; the respiration, which at first was hurried and labored, now becomes stertorous, and accompanied with sighing and moaning; the skin cool, or the surface of the body may retain its natural temperature, though the head may be hot; the sphincters become relaxed; extremities cold; the countenance swollen and livid; the pupils may be dilated, but are often firmly contracted; tracheal râles appear; either the patient is quiet, as if completely paralyzed, or else convulsions, often violent in character, supervene, and he dies suddenly, or he may remain in this condition for several hours.

The first stage corresponds very nearly to that condition described by Southern writers as "solar exhaustion." Dr. Dowler makes a distinction between this "solar exhaustion" (the *coup de soleil* of northern latitudes) and what he calls "solar asphyxia." The former he regards as "a mere fainting, in which the face is pale, skin cool, or not above the natural standard, while, in the latter, the skin is burning hot, face flushed, and the mind and body are utterly insensible to impressions." It runs its course rapidly, and often proves fatal in thirty minutes. Dr. Cartwright says, the cases of "asphyxia are often incurable from falling into an *incurable* state before medical aid can be obtained"! while those of exhaustion simply, if properly treated, will yield as readily as a case of common intermittent, and almost as fatal as "solar asphyxia" if improperly treated.

The second and third stages, described in the progress of the disease, are so intimately connected that it may seem an unnecessary division; but it is more convenient to regard them separately. They differ usually in the mode of attack, and for this reason some have regarded them as a distinct condition. The stage of collapse is most frequently noticed in those who are seized late in the afternoon, "without the signs of apoplexy," after exposure to the heat and fatigue of the day. But the same condition may occur in those who have been seized suddenly "with the signs of apoplexy," and yet pathologically there may be no difference.

Of 60 cases which came under my observation during the past year, 44 were insensible at the time of admission, and 16 were either stupid or insensible. The pupils were dilated in 30 cases, contracted in 19, and natural in 11. The temperature of the body was hot in 34, warm or natural in 14, and cool in 12; while that of the head was elevated in 31, warm in 11, and cool in 18.

The respiration was hurried in 44; the pulse was uniformly accelerated, varying from 100 to 160, and even more per minute. Convulsions were present in 24, delirium was noticed in only a few. Fifty-two of the patients were males. The average duration of the fatal cases was about four hours.

| | | | | | | | | | |
|---|---|---|---|---|---|---|---|---|---|
| The time of the attack in | | | 40 cases was | between | 11 A. M. | and | 4 P.M. |
| " | " | " | " 17 | " | " | " | 4 | | 9 P.M. |
| " | " | " | " 3 | " | " | " | 8 | | 11 A.M. |

Convalescence is usually speedy, after the severity of the disease has passed, and reaction is fully established, varying from a few minutes to five or six hours; the patient sinks into a deep slumber and awakes somewhat exhausted, and the cerebral functions disturbed; but this soon disappears. Two patients only complained of severe pain in the head, and at intervals exhibited great forgetfulness for nearly a week; and one was occasianelly delirious.

A case was reported to me in which delirium supervened, resembling that of delirium tremens. I can conceive that such a condition may exist, but this patient was intemperate, and had been drinking to excess previous to the attack.

Dr. Pepper reports 20 cases, 10 of which died, and 3 resulted in insanity. This termination was not noticed in over 100 cases received at the New York Hospital. In the reports of lunatic asylums, however, few cases of insanity are referable to an attack of *coup de soleil*. One patient was delirious, and with the greatest difficulty restrained.

The statistical reports are too inaccurate to furnish any satisfactory data for the mortality of this disease, as no attempt has been made in the reports to distinguish it from "cerebral apoplexy;" but this latter class is, I believe, less frequently met with than was formerly supposed; and that their number will somewhat diminish as the facilities for *post mortem* examination are furnished, and that by far the greater number of cases included under the head of *coup de soleil* are nothing more than "nervous prostration." About one-half of the cases are usually fatal The mortality of the past year will, however, be above this estimate.

The total number of cases admitted to this Hospital since 1845, is 150, of which 78 died. The mortality of the cases admitted in 1853 is 33 to 67.

The mortality of hospital practice must be greater than that in private, as very many were admitted in a moribund condition, and died before any treatment could be adopted, while others were rendered hopeless by being brought a long distance, several hours after the attack.

The prognosis will depend on the stage of the disease. In the first stage, the prognosis is usually favorable; much, however, will depend upon the treatment adopted. The symptoms indicating collapse are always unfavorable.

In 33 fatal cases, the pupils were contracted in 20, moderately dilated in 7, and markedly so in 6; while, in the successful ones, the pupils were dilated in 18, and nearly natural in 15. No case recovered in which the pupils were contracted. Mere stertorous breathing is not necessarily fatal; but after the respiration becomes *sighing* and *moaning*, the prognosis is very unfavorable; only two patients recovered after this character of the breathing was present.

To these two symptoms—the condition of the pupil and the character of the respiration—I attach much value; and if other observations shall confirm this, they will furnish the most reliable basis for prognosis.

The respiration was sighing or moaning in 31 of the 33 fatal cases; convulsions were noticed in 24. This is a grave symptom, but six recovered after they were present. The pulse alone is no safe criterion of the actual condition of the patient, for it may continue of fair strength throughout the whole course of the disease, with no perceptible alteration either in force or frequency, though the patient may be under the free use of stimulants. This will frequently surprise those who are unaccustomed to observe it.

A fatal relapse occurred in one instance. This patient was attacked suddenly while at his work, and lost all consciousness. As soon as he had sufficiently recovered, he walked a long distance to the Hospital, exposed to the direct influence of the sun. This exertion, combined with his previous prostrated condition, probably induced another attack. He again partially convalesced but immediately sank into a comatose condition, from which he did not rally.

The pathology of this disease is uncertain. We have as yet failed to discover any satisfactory lesion to account for the phenomena noticed before death. It is now, however, generally admitted to be merely "exhaustion" produced by fatigue—either in the sun, or, less frequently, in a close and over-heated apartment.

The post mortem appearances, though of a negative character, are precisely opposite those found in "congestion" of the brain or apoplexy produced by insolation—in other words, *coup de soleil.* And it is of great importance that this relation should be correctly understood, for they obviously require an opposite course of treatment. Unfortunately these two conditions are too indiscriminately called *coup de soleil.* Our nomenclature, in this respect, is imperfect, and calculated to mislead those who are unaccustomed to observe it. But we must not infer, simply because a disease has been erroneously called *coup de soleil*, that we have apoplexy to contend with. "It is debility we have to meet, and not depletion." Depletion, which is essential in the one, is almost necessarily fatal in the other.

In some cases we have apoplectic symptoms with those which properly belong to the opposite condition. And we may perhaps be puzzled to know to which class they belong. But even in these cases, we rarely find any lesion. Sometimes there will be found a moderate congestion of the brain, but no more so than we often find in cases where we suspect no lesion of that organ.

The diagnosis of those cases, which simulate apoplexy is often difficult. The remarks of Dr. Condie, though inapplicable to the case just given, may perhaps be generally useful. He says: "In those cases requiring depletion, the head particularly, and often the entire surface of the body, is hot. The eyes injected; pupils contracted; pulse small, quick, and corded. Tongue red and dry. Patients are delirious, restless, and in a constant state of agitation; and if not speedily relieved by prompt and active treatment coma ensues, and the patient dies as in acute meningitis."

The true pathology of this disease, like those cases of death produced by lightning, will probably never be correctly explained, unless, perhaps, the microscope may aid in removing the veil of mystery which surrounds it. But it must be remarked *en passant*, that there are many points of resemblance in the appearance of those who have died from the effects of heat, and the cases reported of death from lightning.

Does the heat produce death by destroying the "vital principle," as Hunter supposed was the effect of lightning? Does it produce some chemical change in the blood itself, so that it can no longer subserve the purposes of innervation? Or does it produce its effect primarily upon the nervous system? This is the most plausible theory. The vital powers, already enfeebled by fatigue and the heat of the atmosphere, are unduly stimulated. The natural balance of the circulation is destroyed, and the heart contracts with a "morbid activity." The lungs are engorged with blood, and the heart labors to overcome the increased obstacle, until at length it is exhausted by this "morbid activity," and passive congestion takes place in the capillaries throughout the body.

The pathology of this disease is too obscure and uncertain, and observation too limited, to arrive at any satisfactory conclusions in regard to the treatment. It is at best empirical. We regard the disease as one of debility, and we partially treat it as such.

The great practical point to be regarded in the treatment is, that this affection is entirely distinct from *coup de soleil*, as generally understood by the term. It is a disease of "debility," and not one of "repletion." Depletion is generally contra-indicated, and stimulants are usually required.

In cases of *Insolation*, the lance is often employed. But these are very rare. During the summer of 1818, there were 13 cases admitted into the Hospital. These were largely bled; 60 ounces were taken from the arm by repeated bleedings; and in one case as many as 80 ounces. And the "recovery in this one was much more marked and speedy." Three of these died, and the post-mortem appearances were precisely those of "cerebral congestion." But in cases of *exhaustion*, I have never seen a *patient recover* after he had been bled.

This practice is now nearly abandoned. Formerly, nearly every case treated before admission to the Hospital had been bled. But not a *single patient* had been bled of those admitted during the past summer. They do not bear well even the local abstraction of blood by cupping.

The plan of treatment usually adopted is to place the patient in a hot bath, rendered stimulating perhaps by mustard or capsicum—or counter-irritation to the whole body by means of mustard; a stimulating enema of tr. aloes c., or, what is preferable, spts. terebinth; ice to the head *when the temperature is elevated;* brandy and tr. capsici, or even carb. ammonia if required.

The indiscriminate use of *cold affusions* is productive of harm. Injurious and often fatal effects result from them. It is a popular and erroneous idea that a patient, as soon as he is attacked, should be completely deluged with cold water. To employ it in every case would be as absurd as in cases of collapse from any cause.

Another important consideration in the treatment of the earlier stages is *rest*. In crowded cities, to which this disease is mostly confined, this caution is too much disregarded. As soon as a patient is attacked, he should be placed in a horizontal position, in as cool a place as possible, and perfect rest required. Nothing can be more serious for a patient in this condition, than to be carried, as is too often the case, upon an ordinary cart for a long distance, or allowed to remain exposed to the influence of the sun."

